# Interfacial Defect Passivation by Molecular Dipole‐Moment Engineering in Perovskite Solar Cells

**DOI:** 10.1002/smsc.70369

**Published:** 2026-08-26

**Authors:** Jonathan L. Adamu, Somayeh Gholipour, Mayank Kedia, Weiwei Zuo, Asfaw Negash, Goutam Paul, Seyma Topcu, Jose J. Jeronimo‐Rendon, Yunshan Wang, Roberto Félix, Chittaranjan Das, Stefan A. L. Weber, Bettina V. Lotsch, Michael Saliba

**Affiliations:** ^1^ Institute for Photovoltaics (ipv) Research Center SCoPE and Integrated Quantum Science and Technology Center (IQST) University of Stuttgart Stuttgart Germany; ^2^ Nanochemistry Department Max Planck Institute for Solid State Research Stuttgart Germany; ^3^ Department Interface Design Helmholtz‐Zentrum Berlin für Materialien und Energie GmbH (HZB) Berlin Germany; ^4^ IMD‐3 Photovoltaics Forschungszentrum Jülich Jülich Germany

**Keywords:** dipolar molecule, interfacial dipole moment, passivation, perovskite photovoltaics

## Abstract

The performance of perovskite solar cells (PSCs) is critically dependent on the quality of interfaces and grain boundaries, which often lead to nonradiative recombination losses, degradation, and inefficient charge transport. Here, we introduce an asymmetric bulky cation with a dipole moment (2.48 Debye), 4‐methoxyphenethylammonium bromide (MPB), at the perovskite/hole transport layer (HTL) interface in planar (n‐i‐p) PSCs. The MPB provides dual functionality, not only as a defect passivation agent for undercoordinated Pb^2+^ but also improving charge extraction at the perovskite/HTL interface. The MPB‐passivated PSCs exhibit enhanced crystallinity and an increase in hole‐carrier diffusion length (from 540 to 770 nm). We quantitatively attributed the built‐in  potential increase (from 1.09 ± 0.01 to 1.12 ± 0.01 V) to a larger charge‐carrier driving force in MPB‐treated devices, as determined by Mott–Schottky analysis. The optimized PSCs achieve a higher power‐conversion efficiency (PCE) of 22.7% compared to the control device (PCE of 21.5%). Consequently, the MPB‐treated device retained 80% of its initial PCE, compared with 60% for the control, under standard illumination, ~35% relative humidity (RH), and a temperature of ~45 °C after 2000 s. This work provides valuable insights into defect passivation through dipole‐assisted molecular interface engineering, which enhances charge extraction and overall device performance, offering a pathway for high‐efficiency PSCs.

## Graphical abstract

A bulky cation material with a dipole moment, 4‐MeO‐PEABr, is applied to improve interfacial contact and passivate the defects of perovskite films. The molecular dipole moment of the molecule affects the work function of the perovskite film, yielding an efficiency of 22.7% (SPO at 22.5%), and improved performance retention under illumination.

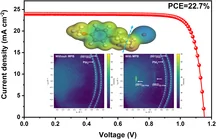

## 1 Introduction

Perovskite solar cells (PSCs) have garnered considerable interest due to their exceptionally high absorption coefficient, low exciton‐binding energy, outstanding optoelectronic performance, potential for low‐cost fabrication [1, 2, 3, 4, 5], and widely tunable bandgaps [6], leading to certified efficiencies of 28.0% for single‐junction solar cells [7, 8, 9, 10]. Bottlenecks, such as surface defects at the perovskite interfaces, hamper further improvements. The interfaces in PSCs are reported to have high‐density trap states, which facilitate nonradiative recombination losses, impair charge transport, significantly limit efficiency, and compromise long‐term operational stability [11, 12].

Therefore, interface engineering via robust defect passivation has become a crucial strategy for enhancing photovoltaic performance. Currently, two‐dimensional (2D) perovskite structures, in which corner‐sharing [BX_6_] octahedra are separated by bulky organic spacer cations into a thin molecular layer, have emerged as attractive passivation materials for the three‐dimensional (3D) perovskite surfaces [13]. Owing to their structural and long‐term exposure tolerance, the resulting 2D/3D heterostructures mitigate some of the challenges under operational conditions, such as moisture and oxygen [14, 15]. Among them, organic ammonium salts, particularly phenethylammonium iodide (PEAI), have attracted attention due to their ability to neutralize trap states (forming quasi‐2D (PEA)_2_PbI_4_ perovskite) and consequently improve device performance [16]. Nonetheless, environmental operating conditions continue to pose a risk to the viability of PSCs in commercial applications [17, 18, 19, 20, 21]. The composition and moiety of these bulky organic cations [22] can significantly influence the interfacial energetics, notably upward band bending, efficient charge‐carrier transport processes, and optoelectronic properties [14].

Previous studies, for example, by Hao et al., have demonstrated that the dipole moment of 2D layers can reduce exciton‐binding energy, significantly increase the work function (WF), the built‐in electric field (*V*_bi_), and improve charge extraction, thereby improving the perovskite device performance [14]. Similarly, a rationally designed 2D layer using 4‐(trifluoromethyl) benzamidine hydrochloride (TFPhFACl) has been shown to have good stability of PSCs (with no decrease in power‐conversion efficiency [PCE]) after storage in a dry‐box for 1500 h below 20% humidity via dipole alignment and defect passivation [23]. Li et al. introduced the concept of 2D/3D heterojunctions using PEA^+^ cations, significantly improving surface passivation, phase stability, and moisture resistance [24]. They also reported that longer‐chain cations, such as 4‐(aminoethyl) pyridine (4‐AEP), form a quasi‐2D phase at grain boundaries with different *n*‐values, offering tunable passivation mechanisms [24]. Last, halide anions (X^−^ = Cl^−^, Br^−^, I^−^) in 4‐MeO‐PEA–X can be employed to modulate the WF via an interface dipole moment, driven by differences in ionic radius and polarizability [25]. Among these halides, I^−^ induces the strongest interfacial dipole and largest downward surface‐WF shift, thereby facilitating hole extraction in p‐i‐n PSCs. By modulating WF while ensuring interfacial stability and mitigating ion migration, Br^−^ demonstrates an optimal tradeoff, while Cl^−^ has a negligible effect on the dipole moment. However, studies focusing on asymmetric bulky cations specifically, those capable of inducing significant dipole moments, remain scarce. For example, methoxy functionalized phenethylammonium cations, such as 4‐MeO‐PEABr (MPB), possess excellent electron‐donating characteristics, including the ability to induce interfacial band bending. This increases the electron density on the benzene ring, facilitating electrostatic interactions with undercoordinated Pb^2+^ ions and effectively passivating surface defects [25]. The presence of ordered dipolar molecules plays a critical role in shifting energy bands and enhancing charge‐transport efficiency [26, 27]. These results suggest that molecular dipoles modulate interfacial band‐bending. Hence, the perovskite modified by MPB with a large dipole moment [25] revealed energy levels compatible with the hole transport layer (HTL), facilitating hole extraction and transport [28]. To our knowledge, interfacial modification using MPB passivation in n‐i‐p perovskite device structures has not been thoroughly explored [29, 30, 31, 32], especially its role in defect passivation, quasi‐2D interfacial formation, and creating preferential charge‐transfer paths at the perovskite/HTL interface. The intrinsic molecular dipole‐induced moment is thought to play an additional role to the experimentally confirmed effective defect passivation and decrease in nonradiative recombination losses.

This study investigates how the molecular dipole moment introduced by the asymmetric bulky cation MPB, used as a top passivation layer on perovskite, enhances charge extraction at the perovskite/HTL interface in n‐i‐p PSCs. The passivated perovskite film exhibits reduced surface roughness, decreasing from 21 to 19 nm and lower defect densities, decreasing from 0.46 × 10^16^ to 0.37 × 10^16^ cm^−3^ compared to the control. The device with MPB achieved a champion PCE of 22.7% and a stabilized power output (SPO) of 22.5%. The unencapsulated device retained about 80% of its initial PCE after 2000 s under standard illumination, 35% relative humidity (RH), and a temperature of 45 °C. Therefore, our results demonstrate that a comprehensive understanding of interfacial charge‐carrier dynamics mediated by bulky dipolar cations is essential to improve both efficiency and stability of PSCs. These findings provide insights into charge‐extraction mechanisms and support the design of passivation materials for scalable, high‐efficiency perovskite photovoltaic technologies.

## 2 Results and Discussion

### 2.1 Molecular Design and Charge‐Extraction Pathway

**FIGURE 1:**
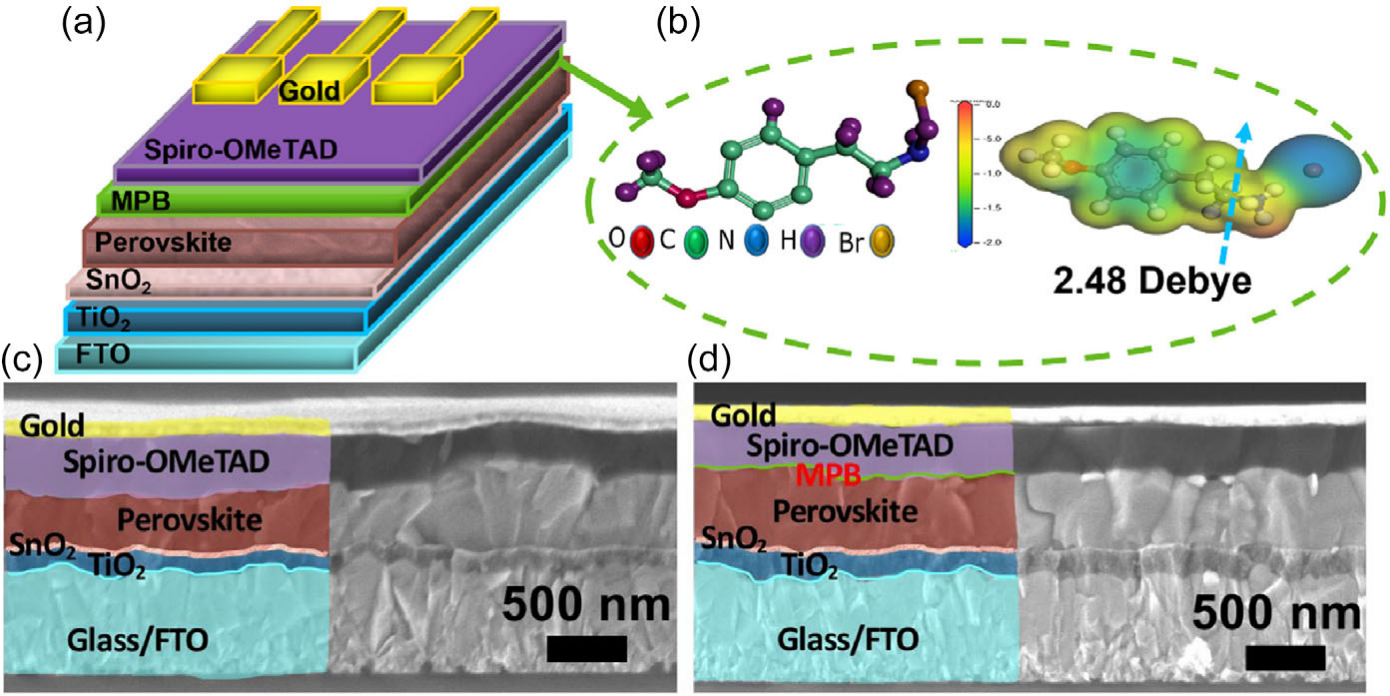
(a) Schematic illustration of the device architecture, (b) chemical structure and ESP mapping image of the MPB cation, and cross‐section SEM images of the devices (c) without and (d) with MPB. Scale bars are 500 nm for both images.

The MPB molecule comprises three key functional components: an electron‐donating methoxy group (−OCH_**3**_), a benzene ring, and an ammonium cation (−NH_3_^+^) paired with a bromide anion (Br^−^). Figure 1a–d illustrates the device integration, molecular structure of MPB, its electrostatic potential (ESP), and cross‐sectional scanning electron microscopy (SEM) images of PSCs, respectively [1]. The perovskite device configuration is introduced in Figure 1a; the ESP map (see Figure 1b) reveals distinct hole‐rich and hole‐deficient regions, arising from the electron‐donating nature of the methoxy group. This also contributes to an asymmetric charge distribution, generating a net dipole moment of 2.48 Debye within the molecule [25]. This polar moiety induces a localized dipole moment, creating a hole‐rich zone near the −OCH_3_ group and a complementary electron‐accumulation region adjacent to the perovskite interface. Conversely, in electron‐depleted areas, the dipole field electrostatically blocks electron migration, thereby promoting selective hole transport [33].

### 2.2 Optoelectronic Properties and Recombination Dynamics

**FIGURE 2:**
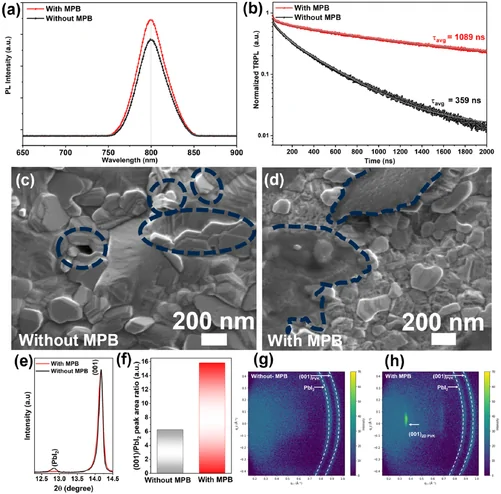
(a) Steady‐state PL, (b) TRPL spectra, (c,d) scanning electron microscopy (SEM) top‐view images, (e) X‐ray diffraction (XRD), (f) (001)/PbI_2_ peak area ratio, and (g,h) grazing‐incidence wide‐angle X‐ray scattering (GIWAXS) analysis for the perovskite films with and without MPB surface treatment.

Spectroscopic measurements, including UV–vis spectroscopy, steady‐state photoluminescence (PL), and time‐resolved PL (TRPL), are conducted to investigate the influence of MPB surface treatment on the optoelectronic properties of perovskite films. As depicted in the UV–vis absorbance spectra, Figure S1a shows higher absorbance intensity from 600 to 800 nm across the visible spectrum for the MPB‐treated perovskite (with MPB) than the control (without MPB). Moreover, the bandgap calculations from the Tauc plot (Figure S1b) reveal 1.52 eV for both films. Thus, the MPB modification enhances optical properties without compromising the bandgap of perovskite films. As shown in PL spectra (see Figure 2a), both control and MPB‐treated perovskite films exhibit a characteristic emission peak at approximately 800 nm. Moreover, the perovskite film with MPB exhibits an improvement in PL intensity, which is attributed to effective passivation of surface defects by the MPB interlayer. In addition, the quasi‐2D perovskite peak at 475 nm can be detected in the PL spectra (see Figure S1c), where similar trends have been observed in previous reports [34, 35]. The TRPL measurements (see Figure 2b) reveal a notable prolongation in average carrier lifetime (*τ*_avg_) for the sample with MPB, with an average lifetime of 1089 ns, compared to 359 ns for the film without MPB (see Table S1 for details of the average lifetime calculation). The prolonged lifetimes indicate suppressed nonradiative recombination losses and increased fill factor (FF) and open‐circuit voltage (*V*_OC_). We propose that the molecular dipole moment and ordered alignment of MPB molecules result in more effective surface passivation [36, 37, 38].

### 2.3 Morphological and Structural Characterization

To understand the enhanced device performance by MPB treatment, we examined the influence of the molecule on the surface morphology and crystallinity of the perovskite films. Figures 2c,d, and S2a,b present comparative top‐view and cross‐sectional SEM images of perovskite films with and without MPB, respectively. The film without MPB (Figure 2c) exhibits a dense morphology with few whitish particles (see Figure S2c), likely indicative of undercoordinated PbI_2_ [39]. Remarkably, the MPB‐treated sample (Figure 2d) shows the formation of island‐like features on the surface (see Figure S2d). The cross‐sectional views in Figures S2a,b reveal these morphological differences while maintaining comparable film thickness (550 nm). The modified perovskite film (see Figures S2b and 1d) showed coherent grains spanning from the bottom to the top, compared to the control film (see Figures S2a and 1c). This grain structure facilitates efficient charge‐carrier transport. Further investigations of the crystallinity of the control and treated films were conducted by X‐ray diffraction (XRD), as presented in Figure S3. Both films showed dominant reflections (with negligible formation of the photoinactive δ phase) at 14.2° and 28.3°, which are indexed to the desired tetragonal crystalline lattice planes (001) and (002), respectively. Noticeably, the reflections of the lattice planes (001) and (002) of the control film are slightly higher than those of the treated film as a result of the screening effect of the passivation layer. However, as shown in Figure 2e,f, the peak area ratio of (001)/PbI_2_ has increased from 6.2 to 15.8. In addition, the PbI_2_ residues are suppressed after MPB treatment by forming a quasi‐2D perovskite, as seen in (Figure S3) by additional diffraction peaks at 5° (*n = 1*) and 9.7° (*n = 1*) [1, 23, 40]. The observed surface modification suggests a chemical interaction between MPB and residual PbI_2_, potentially forming a coordinated (MeOPEA)_2_Pb(I_(1‐*x*)_Br_*x*_)_4_ that passivates defects and improves film uniformity. This enhancement in structural properties correlates with the improved optoelectronic properties observed in PL and TRPL measurements (see Figure 2a,b).

To gain further insight into the 2D/3D phases on the surface of the perovskite, we performed grazing‐incidence wide‐angle X‐ray scattering (GIWAXS) measurements at an incident angle (*α*) of 0.2°. As shown in Figure 2g,h, both untreated and MPB‐treated films exhibit uniform polycrystalline Debye–Scherrer rings assigned to the PbI_2_ and (001) plane of the 3D perovskite, respectively. The addition of the MPB bulky organic cation entails a sharp and discrete Bragg spot at a low in‐plane scattering vector (*q*_xy_ ≈ 0.35 Å^−1^). This highly oriented spot is indexed to the *n = * 1 (001) plane (5°) of the quasi‐2D perovskite. Furthermore, for the MPB‐treated perovskite, an additional diffraction ring at about *q*_xy_ ≈ 0.72 Å^−1^ is observed, which could be attributed to the *n = *1 (002) phase (9.7°) of the quasi‐2D perovskite [41, 42, 43]. This feature, alongside the distinct *n =* 1 peak, is consistent with the reduction in the intensity of the PbI_2_ diffraction, strongly suggesting that the MPB cation promotes the consumption of the residual PbI_2_ and the subsequent formation of various quasi‐2D perovskite phases. The integrated 1D GIWAXS profile of untreated and MPB‐treated samples (see Figure S4) also confirms the formation of (001) and (002) phases of quasi‐2D perovskite on top of the bulk perovskite.

The observed diffraction pattern indicates an aligned layered structure, suggesting that the bulky organic cation facilitates anisotropic crystal growth with preferential orientation of the layers perpendicular to the substrate. Such alignment is known to facilitate more efficient out‐of‐plane charge transport, which is crucial for enhancing device performance [44]. These findings highlight the complementary role of the molecular dipole moment introduced by MPB in modulating crystallization dynamics. The aligned quasi‐2D perovskite structure not only improves film morphology in terms of pinholes but also creates an unimpeded charge transport pathway across the perovskite layer. These results correlate with a reduction in interfacial recombination (see Figure 2b, TRPL spectra, *τ*_avg_ = 1089 ns for the treated samples with MPB vs. *τ*_avg_ = 359 ns for those without MPB). In addition, systematic optimization of MPB concentration (2, 4, and 6 mg ml^−1^) shows 4 mg ml^−1^ as the optimized surface posttreatment, delivering a champion device with significantly improved UV, PL, and TRPL (see Figure S5a–c for various MPB concentrations).

### 2.4 Photovoltaic Performance and Device Physics

**FIGURE 3:**
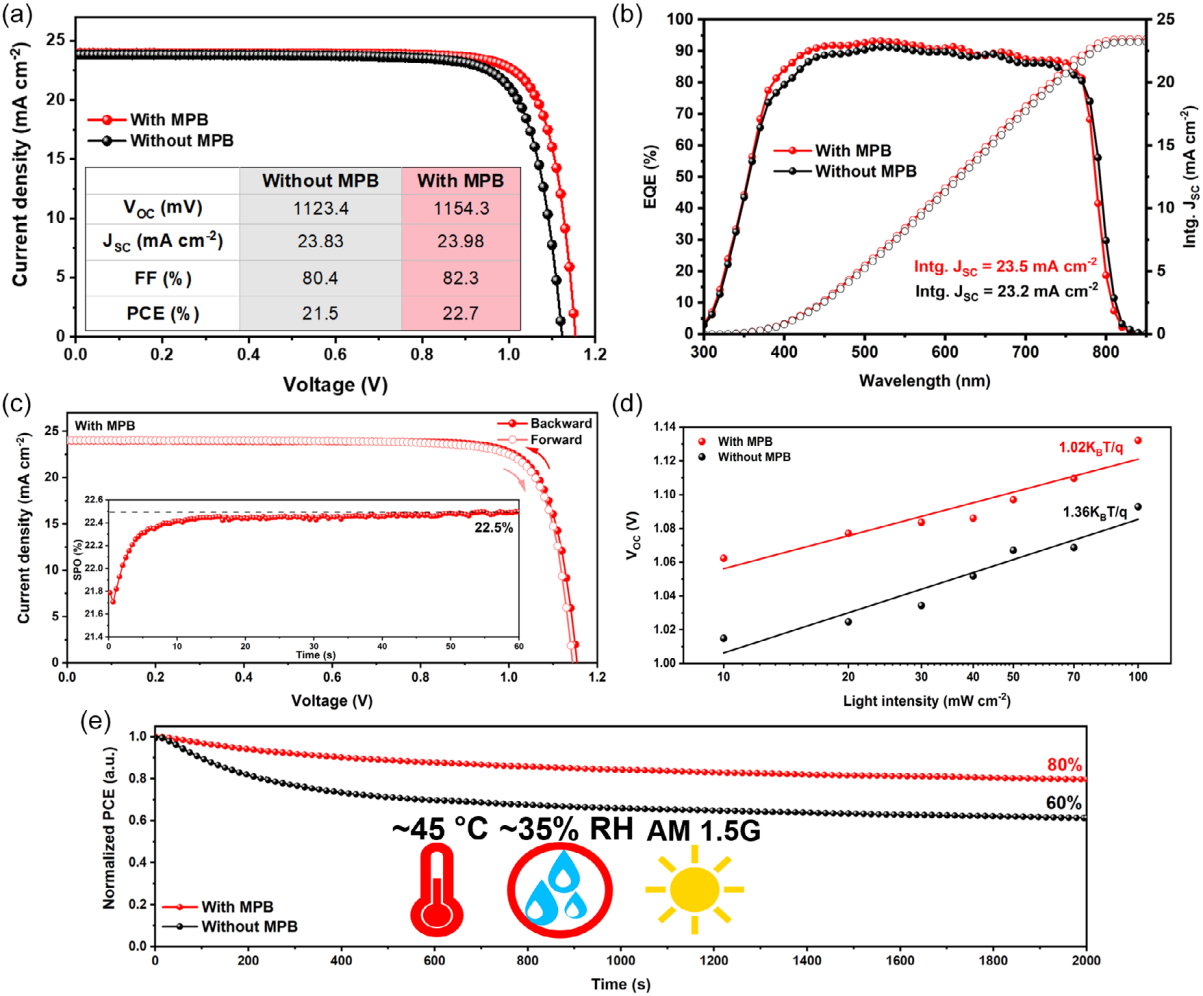
(a) Current density–voltage (*J–V*) characteristics of PSCs with and without MPB, (b) external quantum efficiency (EQE) spectra and integrated *J*_SC_ of PSCs, (c) steady‐state power output and *J–V* characteristics of the champion perovskite solar device in forward and backward scan directions, (d) light intensity‐dependent *V*_OC_ curve for devices fabricated by perovskite films with and without MPB surface treatment, and (e) stability test of unencapsulated device under 35% relative humidity at 45 °C temperature.

The photovoltaic performance of PSCs with different concentrations of the MPB‐treatment is comprehensively summarized in the supporting information (Figures S6 and S7). Moreover, Figure 3a presents the current density–voltage (*J–V*) characteristics of optimized devices with and without MPB under AM 1.5G illumination. The optimized MPB‐treated device exhibited a champion PCE of 22.7%, a *V*_OC_ of 1154.3 mV (30.9 mV absolute increment), FF of 82.3% (1.9% of absolute improvement), and a short‐circuit current density (*J*_SC_) of 23.98 mA cm^−2^. The improvement in PCE of the devices with MPB is attributed to the enlarged FF and *V*_OC_. Comprehensive extracted photovoltaic performance, including forward and backward scan direction, is shown in Figure 3c, demonstrating a minimal hysteresis index of 1.2% (see Table S2). This indicates an outstanding interfacial charge extraction and slight dipole‐enhanced band alignment stabilization under operational bias. This performance improvement stems from two synergistic effects of MPB treatment: defect passivation and supplementary induced dipole moment, comprising the suppression of nonradiative recombination centers at grain boundaries and interfaces, as evidenced by an increase in carrier lifetime and WF, respectively, as shown in Figure 2b.

Notably, the control device showed a *J*_SC_ of 23.83 mA cm^−2^, when compared to the champion device, a lower *V*_OC_ of 1123.4 mV, and FF of 80.4%, yielding a PCE of 21.5%. The result suggests that the dual functionality of MPB as both a passivating agent and dipole modulator, tuning interface energetics and enhancing the performance of planar PSCs [39]. The overall device performance and charge‐collection efficiency are quantified via the integrated *J*_SC_ and the spectral response of the champion device, with external quantum efficiency (EQE) measurements (Figure 3b). The device with MPB exhibits a relatively stable photo‐response across spectral coverage (350–800 nm) with >85% EQE across the visible range (peaking at 580 nm), demonstrating efficient photon‐to‐electron conversion. Integration of the EQE spectrum is in good agreement with *J*–*V* characteristics. It yields an integrated *J*_SC_ of 23.5 mA cm^−2^, matching the derived *J*–*V* measurements (23.98 mA cm^−2^) within 2.0% error, validating measurement consistency and minimal spectral mismatch [45]. The device with MPB is evaluated through the steady‐state power output under continuous illumination (see the inset of Figure 3c). The device exhibited a stabilized PCE of 22.5% over 60 s, indicative of both enhanced film quality and improved operational stability [14]. To further scrutinize the supplementary effect of the dipole molecules on the charge carrier's recombination mechanism, the *V*_OC_ relative to light intensity characterization was performed, in which the calculated slope and its deviation from unity k_B_T/q demonstrate the trap‐assisted recombination in PSCs. In Figure 3d, the slope of the curve for the MPB‐treated device is 1.02 k_B_T/q, which is reduced relative to the control (1.36 k_B_T/q). The lower slope value indicates that the trap‐assisted Shockley–Read–Hall (SRH) recombination is suppressed through the trap passivation of the MPB molecule. Moreover, Figure 3e shows the SPO measurements for devices with and without MPB passivation under standard illumination and ~35% RH and ~45 °C temperature. The results reveal that the sample with MPB starts to saturate at 200 s and retains 80% of its initial performance after 2000 s, while the control device degrades severely, starts to saturate at 400 s, and retains only 60% of its initial PCE under the same conditions. Figure S7a–d and Table S3 compare the statistical distribution of the key photovoltaic parameters (*V*_OC_, *J*_SC_, FF, PCE) for 77 (per condition) fabricated devices of control and MPB‐modified surfaces. The treated devices exhibit a shift in average *V*_OC_, increasing from 1081.0 ± 25.6 mV in the control to 1125.8 ± 16.3 mV in the posttreated devices. The increase in *V*_OC_ suggests that the intrinsic induced dipole moment reduces the energy barrier, thereby improving charge extraction as evidenced in the reduced nonradiative recombination losses and increased carrier lifetime [46], which translates into a corresponding rise in average PCE from 20.0 ± 1.6% to 20.9 ± 0.9%. Additionally, improvement in average FF from 77.6 ± 3.2% to 78.6 ± 2.1% can be attributed to more favorable energy‐level alignment at the perovskite–HTL interface and enhanced contact quality, leading to reduced *V*_OC_ loss. To analyze the mechanism of FF enhancement by MPB treatment, the calculation of maximum FF is conducted (see Equation (S3)). As shown in Figure S8, the improvement of FF is mainly attributed to the reduction in the nonradiative recombination loss and charge‐transport loss.

### 2.5 Mechanistic Insights Into Dipole‐Induced Enhancement

**FIGURE 4:**
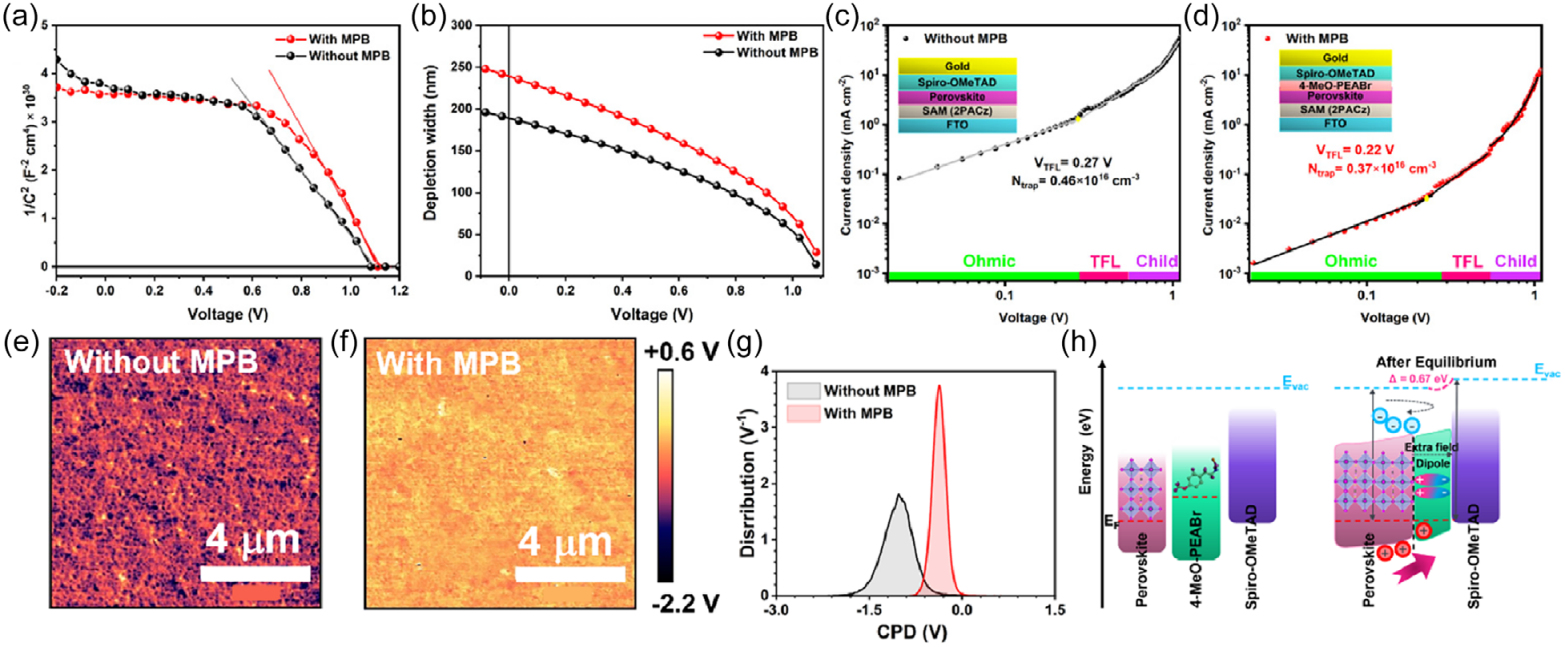
(a) Mott–Schottky curves and (b) depletion width, space‐charge‐ limited current (SCLC) with the hole‐only structure for devices fabricated (c) without and (d) with MPB treatment, Kelvin probe force microscopy (KPFM) images for (e) without and (f) with MPB, and (g) contact potential differences (CPD) distribution from KPFM for perovskite films with and without MPB passivation, and (h) the illustration of the energy‐level alignment before and after equilibrium condition for devices passivated with MPB.

To gain more insight into the effect of the molecules on charge‐carrier dynamics at the interface, we performed capacitance–voltage characterization to visualize the Mott–Schottky plots (Figure 4a,b). The built‐in potential is the main driving force for understanding band bending [47]. The treated devices show an increase in the built‐in potential from 1.09 ± 0.01 V to 1.12 ± 0.01 V (see Figure 4a) and in the depletion width from 189 to 239 nm (Figure 4b) compared to the control. These improvements signify enhanced interfacial energetics and efficient charge separation. Furthermore, by subtly altering the local electrostatic environment and band bending at the perovskite/HTL interface, the inherent molecular dipole of MPB may have contributed to the observed changes [48]. Thus, it effectively dissociates the charge carriers and suppresses the accumulation of charge carriers at the interface [49].

In addition, hole‐only (FTO/SAM(2PACz)/perovskite/Spiro‐OMeTAD/Au) devices (see the inset of Figure 4c,d) and the space‐charge‐limited current (SCLC) method are used to investigate the hole‐diffusion length (*L*_D_), hole‐carrier mobility (*µ*_h_), and density of defects (*n*_trap_). Although the difference in hole mobility is insignificant after MPB treatment, the increase in carrier lifetime results in a rise of the *L*_D_ from 540 to 770 nm, indicating suppressed nonradiative recombination losses [50]. Consequently, the FF enhancement resulted from reduced defect‐assisted recombination and improved interfacial charge extraction rather than mobility enhancement alone [51]. The dark *J–V* curve can be divided into three regions: the ohmic region (*J*∝*V*), the trap‐filling limit region (TFL section, *J*∝*V* ^*n*^, *n* > 2), and the trap‐free Child's‐law region (*J*∝*V* ^2^). The ohmic section of the dark *J–V* curve can deliver the conductivity (*σ*) and the free hole‐carrier concentration (*n*_c_). The switch point between the ohmic and the TFL region is the trap filling limit voltage (*V*_TFL_), where all traps are filled, and the *n*_trap_ can be defined [52]. In the Child's‐law region, the *μ*_h_ can be derived from the Mott–Gurney equation [53]. The results of the analysis are shown in Table S4 (see details in the Supporting Information for calculating *L*_D_, *μ*_h_, and *n*_trap_). Therefore, from Figure 4c,d, it is found that the density of defects (*n*_trap_) is decreased for the perovskite films with MPB (0.37 × 10^16^ cm^−3^) compared to the control film (0.46 × 10^16^ cm^−3^), which implies effective defect passivation in the highly crystalline treated‐perovskite films.

The atomic force microscope (AFM) characterizations indicate that the surface roughness of the perovskite films (RMS height = 19 nm) treated with an optimized concentration of MPB (Figure S9c) is smaller than that (RMS height = 21 nm) of the control film (Figure S9a), which is consistent with the top‐view SEM images (Figure 2c,d). In addition, it can be observed from the AFM images (see Figure S9 and Table S5) that the dipole molecule affects the roughness of the perovskite films. A rougher perovskite surface (25 nm) for a higher concentration of MPB (Figure S9d), compared to the control film (Figure S9a), can be attributed to the preferential accumulation of MPB at the grain boundaries and the perovskite surface, altering nucleation and crystal growth dynamics after posttreatment, which resulted in a slight increase in the topographical variations as detected with AFM [54].

Therefore, the lower roughness of the treated film compared to the control subtly improves the interfacial contact with the subsequent HTL, which becomes more hole‐conductive to carrier transport, and this also contributes to the enhanced efficiency observed in MPB‐treated devices.

To further validate our investigation, we performed Kelvin probe force microscopy (KPFM) measurements to investigate the perovskite/HTL interface in more detail for both films (see Figure 4e,f). As shown in Figures 4g, S9, and Table S5, the MPB‐treated film demonstrates a narrower distribution and a higher average contact potential difference (CPD) compared to the control. A dipole orientation with the negative side pointing upward results in a lower potential. Conversely, an upward‐pointing positive side leads to a higher CPD. In this regard, the MPB‐treated film exhibits a higher CPD value compared to the control sample, which may be attributed to the desired dipole moment. While this increase in CPD is consistent with a possible contribution from the intrinsic molecular‐induced dipole moment of MPB, it primarily arises from the reduced surface defect and the formation of a quasi‐2D interfacial layer. Besides, the perovskite films with MPB exhibit a lower CPD variation than the control film, as shown in Figure 4g. Moreover, the KPFM images corresponding to different concentrations of MPB (0, 2, 4, 6 mg ml^−1^) are shown in Figure S9a–d, revealing patches in the topography of the sample with 4 and 6 mg ml^−1^ MPB, while the sample with 4 mg ml^−1^ MPB exhibits enhanced surface uniformity. These observations indicate the formation of thicker self‐assembled monolayers (SAMs) for the sample with MPB (4 mg ml^−1^) than the control one, which results in a decrease in the surface potential difference between grain interior and grain boundaries. Nonetheless, an increase in MPB concentration to 6 mg ml^−1^ leads to accumulation at the surface, likely resulting in partial aggregation and nonuniform SAM coverage, and thereby increasing surface roughness (~25 nm). These findings may be attributed to reduced surface defects and improved film quality for the sample with MPB, resulting from the coordination effect at the film interface. Based on the above investigations, we propose a possible mechanism for how the surface electric dipole layer induced by MPB modulates the energy levels at the perovskite/HTL interface, as shown in Figure 4h. The corresponding energy level diagram (Figure 4h) shows that MPB treatment increases the WF of the perovskite layer by 0.67 ± 0.38 eV, where the WF difference between control and MPB‐treated perovskite films is calculated by subtracting the measured average CPD values of the two films (Table S5) [55]. These changes are attributed to the inherent net dipole moment of the quasi‐2D perovskite, arising from its electron‐donating —OCH_3_ group at the para position, which induces an upward vacuum level shift (Δ = 0.67 ± 0.38 eV). Such a change leads to favorable modification at the perovskite/HTL interface, which promotes carrier transport by increasing the driving force and thus suppresses nonradiative recombination at the interface, thereby accounting for the overall enhancement of device performance, which is comparable to the previous reports on the application of dipole‐moment passivation agents in n‐i‐p PSCs (see Table S6).

Furthermore, time‐of‐flight secondary ion mass spectrometry (TOF‐SIMS) analysis was conducted to analyze the depth‐profile distribution of the 4‐MeO‐PEA^+^ cations. As shown in ion depth profiles and 3D topographic images (Figure S10), 4‐MeO‐PEA^+^ ions are primarily distributed at the upper interface and grain boundaries of the perovskite films. This aligns with the characteristic emission peaks of quasi‐2D perovskite as detected by XRD (see Figure S3). The combination of TOF‐SIMS and XRD indicates that quasi‐2D perovskites may be distributed on the surface and the grain boundaries of 3D perovskite films.

**FIGURE 5:**
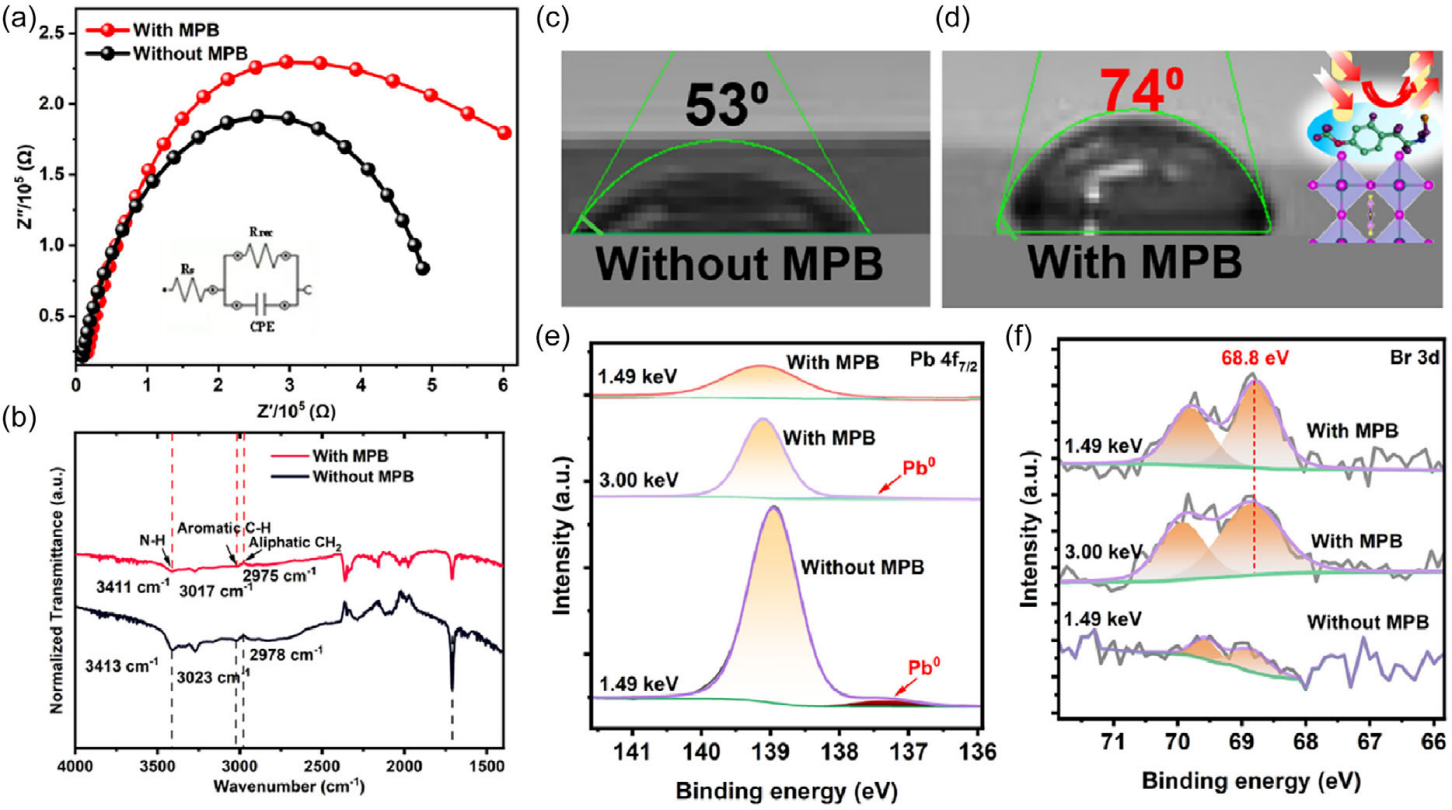
(a) EIS, (b) FTIR, (c) and (d) contact angle, XPS spectra of (e) Pb 4f and (f) Br 3d for the perovskite films without and with MPB treatment.

To verify the interfacial charge recombination dynamics, electrochemical impedance spectroscopy (EIS) measurements are conducted on both devices with and without MPB treatment in the dark at a bias of 0.6 V. The Nyquist plots shown in Figure 5a indicate that the device with MPB has a higher charge recombination resistance (*R*_rec_) of 6.19 × 10^5^ Ω, compared to the control device *R*_rec_ of 3.44 × 10^5^ Ω. These results suggest that the device with MPB significantly improves carrier transport and suppresses interfacial recombination, leading to lower energy losses and better overall device performance. Furthermore, the interaction between MPB and the perovskite film has been further confirmed by Fourier transform infrared (FTIR) spectroscopy. As shown in Figures 5b and S11, a subtle stretching vibration peak of N—H at 3413 and bending vibration of N—H at 662 cm^−1^ for the control perovskite films redshifts to 3411 and 656 cm^−1^ for the MPB‐passivated film, indicating the interaction between them by the formation of a hydrogen bond of MPB ammonium to surface halides [25]. In addition, aromatic C–H at 3023 cm^−1^ and aliphatic CH_2_ at 2978 cm^−1^ redshifted to 3017 and 2975 cm^−1^ in the presence of MPB interaction. In addition, the control perovskite film shows a contact angle of 35° (see Figure 5c), reflecting its relatively polar and defect‐rich surface. After MPB passivation, the contact angle increases to 74° (see Figure 5d). This enhancement in hydrophobicity arises from the outward‐oriented aromatic and methoxy substituents of the MPB cation. The hydrophobic MPB layer protects the perovskite against moisture and confirms successful surface coverage, which is verified by better stability measurements under 35% RH (see Figure 3e).

Moreover, the interaction between the passivating molecules 4‐MeO‐PEA^+^ and ions on the perovskite surface is then studied through X‐ray photoelectron spectroscopy (XPS) as shown in Figure 5e,f. In Figure 5e, the control sample displays the Pb 4f_7/2_ peaks at binding energies 138.9 and 137.3 eV related to the Pb^2+^ state in perovskite and unreacted metallic Pb [56, 57]. It is further revealed by the decreased Pb^0^ component in perovskites after interfacial modification, whose deconvoluted peaks centered at lower binding energies compared to the lattice Pb with the most intense signals (Figure 5e). The suppression of the Pb^0^ signal at excitation energy of 1.49 keV after MPB treatment indicates that MPB effectively passivates undercoordinated PbI_2_ sites at the surface. The deeper‐probing 3.00 keV spectra show minimal Pb^0^ even without MPB, confirming Pb^0^ is a surface‐localized defect. Therefore, the Lewis acid–base interaction between the residual PbI_2_ and the 4‐MeO‐PEA^+^ leads to the transfer of the electron cloud related to the 4‐MeO‐PEA^+^ ion and quasi‐2D perovskite phase formation. As depicted in Figure 5f, the Br 3d core level at a binding energy of 68.8 eV associated with perovskite shows lower intensity in the control sample [58, 59]. In contrast, it is significantly increased for both excitation energies, 1.49 and 3.00 keV, in the MPB‐treated film, confirming that the MPB interacts with the perovskite and subsurface region. As shown in Figure 5f, the Br 3d core level intensity, attributed to bromide in the perovskite as well, is significantly higher at an energy of 1.49 keV compared to 3.00 keV for the MPB‐treated film, confirming that the MPB molecule remains localized at the surface, rather than diffusing into the bulk.

## 3 Conclusion

Our study revealed the combined passivation effects of molecular dipole‐induced interfacial modification on the WF of the perovskite film and the photovoltaic device performance. Additionally, the molecular methoxy‐functionalized cations create favorable energy‐level alignment, causing an upward shift in the perovskite WF and an enhanced built‐in potential, leading to improved *V*_OC_. It is suggested that the strong bonding between the methoxy group in MPB and the undercoordinated Pb^2+^ defects in perovskite contributes to the longer carrier lifetime in perovskite films treated with MPB. The combined effects resulted in the perovskite modified with MPB presenting the champion device with a PCE of 22.7% (SPO at 22.5%) and improved storage and illumination stability. Our work provides valuable insights into the molecular dipoles at interfaces for tuning device performance.

## 4 Experimental Section

### 4.1 Materials

Lead iodide (PbI_2_, 99.9%), cesium iodide (CsI, 99.999%), formamidinium iodide (FAI, 99.9%), methylammonium chloride (MACl), methylammonium bromide (MABr), 4‐methoxyphenethylammonium bromide (4‐MeO‐PEABr, >99%), dimethyl sulfoxide (DMSO > 99.9%), dimethylformamide (DMF 99.8%), chlorobenzene (CBZ, 98%), ethanol (anhydrous, 0.01 H_2_O), titanium diisopropoxide bis(acetylacetonate), 2,2′, 7,7′‐tetrakis[*N*,*N*‐di(4‐methoxyphenyl)amino]‐9,9′‐spirobifluorene (Spiro‐OMeTAD), lithium bis(trifluoromethanesulfonyl)imide (Li‐TFSI), and FK209 Co(III) complex were purchased from Sigma–Aldrich and Great Solar Cell Ltd, respectively. [2‐(9H‐carbazol‐9‐yl)ethyl]phosphonic acid (2PACz) was purchased from Dyenamo AB. Gold nuggets were used for the top electrode. All chemicals were used as received, without further purification.

### 4.2 Device Fabrication and Thin‐Film Preparation

FTO glass substrates (25 × 25 mm, 15 Ω/sq.) were sequentially cleaned using Hellmanex solution (2% V/V) and deionized water in an ultrasonic bath, followed by ultrasonic cleaning in acetone and isopropanol for 15 min. The substrates were dried using a nitrogen stream. A compact TiO_2_ layer was deposited on FTO by aerosol spray pyrolysis using oxygen as the carrier gas. The precursor solution was prepared by diluting 0.6 ml of titanium diisopropoxide bis (acetylacetonate) (75 wt.% in isopropanol) in 10 ml of anhydrous ethanol. The substrates were preheated to 450 °C and maintained at this temperature for 15 min before and 30 min after spraying. The TiO_2_‐coated substrates were treated with UV‐ozone plasma for 30 min, followed by dissolving 0.019 g of tin(II) chloride dihydrate in 1 ml anhydrous ethanol (mix inside the glove box), then spin‐coated as a SnO_2_ electron‐transport layer (ETL) at a spin coating speed of 3000 rpm, 1000 rpm s^−1^ for 30 s. These substrates were then annealed at 200 °C for 60 min. The perovskite triple cation precursor solution Cs_0.04_(FA_0.85_MA_0.11_)Pb(I_0.96_Br_0.01_Cl_0.03_)_3_ was prepared with a molarity of PbI_2_ (1.35 M), FAI (1.12 M), MABr (0.05 M), MACl (0.10 M), and CsI (0.05 M), which were dissolved in DMF: DMSO (3:1, V:V). The prepared solution was stirred overnight on an automatic vortex stirrer. The perovskite film was deposited using a two‐step spin‐coating method after a second 30‐min UV‐ozone plasma treatment. Spin parameters were as follows: 1000 rpm with an acceleration of 100 rpm s^−1^ for 12 s (spread step) and 4000 rpm with an acceleration of 1000 rpm s^−1^ for 25 s (spin step). Chlorobenzene (300 µl) was used as an antisolvent and dripped 10 s before the end of the spin‐coating process. The films were annealed at 100 °C for 40 min in a nitrogen‐filled glove box. The passivation solution was prepared by dissolving different concentrations (0, 2, 4, 6 mg ml^−1^) of 4‐MeO‐PEABr in anhydrous isopropanol (IPA). The passivation solution was dynamically deposited onto the cooled perovskite film by spin coating at 4000 rpm, 1000 rpm s^−1^ for 30 s, followed by annealing at 100 °C for an additional 5 min. The Spiro‐OMeTAD HTL was prepared by dissolving 92 mg in 1 ml chlorobenzene, adding 20.9 µl of Li‐TFSI solution (520 mg in 1 ml acetonitrile), 36.3 µl of tBp, and 15 µl of FK‐209 (150 mg in 400 µl of acetonitrile). The HTL was spin‐coated in two steps: Step 1: 100 rpm, for 2 s and Step 2: 1800 rpm for 28 s, both at 1000 rpm s^−1^ acceleration. Finally, to complete the device fabrication, a 100‐nm gold (Au) electrode was deposited via thermal evaporation under high vacuum (<5 × 10^−6^ Torr) via a shadow mask that defines the active area of the devices (0.16 cm^2^). For the SCLC measurement, FTO substrates were coated with 2PACz SAM (0.5 mg ml^−1^ in anhydrous ethanol) by spin coating at 3000 rpm, 1000 rpm s^−1^ for 30 s.

### 4.3 Characterization and Measurements

#### 4.3.1 Characterization of Device Performance

Current density–voltage (*J–V*) measurements were conducted using a Keithley 2400 source meter under AM 1.5G illumination (100 mW cm^−2^) (WAVELABS SINUS‐70) solar simulator. Measurements were taken in ambient conditions using a 0.089 cm^2^ optical mask in both forward and reverse scan directions from −0.2 to 1.2 V at a scan rate of 20 mV s^−1^. Devices were measured within 1 week after fabrication to assess stability and hysteresis behavior. The SCLC measurement for a hole‐only device was done with a Keithley 2400 (at a scan rate of 5 mV s^−1^).

##### 4.3.1.1 SPO

The SPO of the devices was measured by monitoring the output current for 60 s.

##### 4.3.1.2 Stability Test

The performance of the PSCs was assessed using a Keithley 2400 source meter at 45 °C, 35% RH, under AM 1.5G solar irradiation.

##### 4.3.1.3 EQE

EQE spectra were measured using an Enlitech QE‐R system equipped with a monochromator and a calibrated silicon photodetector.

##### 4.3.1.4 EIS

Impedance spectroscopy measurements were conducted using an Autolab potentiostat (PGSTAT302N) at a bias voltage of 0.8 V under dark conditions, with a frequency range from 1 mHz to 1 MHz and a voltage perturbation amplitude of 20 mV.

#### 4.3.2 Optoelectronic Characterizations

##### 4.3.2.1 UV–Vis–NIR Spectroscopy

Reflection and transmission spectra were recorded using a PerkinElmer Lambda 1300 spectrometer equipped with an integrating sphere.

##### 4.3.2.2 Steady‐State and Time‐Resolved PL Spectroscopy

Measurements were performed using a custom‐built system.

#### 4.3.3 Structural and Morphological Characterization

##### 4.3.3.1 SEM

SEM images were obtained using a JEOL JSM‐IT800 scanning electron microscope in a high vacuum using beam deceleration mode.

##### 4.3.3.2 XRD

Structural properties were analyzed using an X'PertPro (PANalytical) diffractometer in Bragg–Brentano geometry with Cu Kα_1_ radiation (*λ* = 1.5406 Å), scanned from 5° to 50° (2*θ*).

##### 4.3.3.3 GIWAXS

Synchrotron‐based XRD for the GIWAXS was carried out at beamline KMC‐2 at BESSY II, HZB, Germany. The instrument was equipped with a Bruker Vantec 2000 area detector. The X‐rays had an energy of 8 keV, optimized for the beamline, and XRD patterns were collected at 300 K. For the GIWAXS measurements, an incident angle (*α*) of 0.2° was used. The 2D images were plotted using GADDS software, and the scattering vector (*q*) was calculated during postimage processing using an in‐house‐developed Python script.

#### 4.3.4 Surface and Chemical Characterization

##### 4.3.4.1 XPS

The X‐ray photoelectron spectroscopy measurement was done with two different photon energies of 1.49 keV, the laboratory‐based X‐ray, and 3.00 keV at the synchrotron light source. The laboratory‐based XPS measurement was done with an Al *K*_α_ monochromatic source. The hard XPS (HAXPES) measurements were done at the HiKE endstation [60], located at the BESSY II KMC‐1 beamline [61] at HZB, using a Scienta R4000 electron analyzer. All the data were corrected with respect to the Au4f measured at the respective measurement systems.

##### 4.3.4.2 KPFM

The AFM and KPFM were carried out on Asylum Research MFP3D operating in N2 by tapping mode (Multi75E‐G tip, *k* = 3 N/m, Pt coating).

##### 4.3.4.3 FTIR

FTIR was performed by the iS50 FT‐IR (Nicolet).

##### 4.3.4.4 TOF‐SIMS

A liquid metal ion gun with a 30 keV Bi beam was used as the analyzing source, in an area of 50 × 50 µm^2^. A sputtering source of a metal gun with a Cs gun was used as a secondary source, operating at 2 keV with a sputter crater of 200 × 200 µm^2^. The sample was measured without light to avoid charging effects. The detection was done in spectrometry mode and with positive polarization.

## Conflicts of Interest

The authors declare no conflicts of interest.

## Supporting information

Supplementary Material

## Data Availability

The data that support the findings of this study are available from the corresponding author upon reasonable request.
