# Supplementary material for: Interfacial Defect Passivation by Molecular Dipole‐Moment Engineering in Perovskite Solar Cells
Source: Small Sci. 2026 Aug 26;6(9):e70369. doi: 10.1002/smsc.70369 (PMC13516869; doi:10.1002/smsc.70369)
Supplement: Supplementary file 1 [file SMSC-6-e70369-s001.pdf]

SUPPORTING INFORMATION

Interfacial Defect Passivation by Molecular Dipole Moment Engineering in Perovskite Solar Cells

Jonathan L. Adamu, Somayeh Gholipour, Mayank Kedia, Weiwei Zuo, Asfaw Negash, Goutam Paul, Seyma Topcu, Jose J. Jeronimo-Rendon, Yunshan Wang, Roberto Félix, Chittaranjan Das, Stefan A. L. Weber, Bettina V. Lotsch, and Michael Saliba**

J. L. Adamu, S. Gholipour, M. Kedia, W. Zuo, A. Negash, G. Paul, S. Topcu, Y. Wang, C. Das, S. A. L. Weber, M. Saliba

Institute for Photovoltaics (*ipv*), Research Center SCoPE, and Integrated Quantum Science and Technology Center (IQST), University of Stuttgart, Stuttgart, Germany

E-mail: somayeh.gholipour@ipv.uni-stuttgart.de; michael.saliba@ipv.uni-stuttgart.de

M. Saliba

IMD-3 Photovoltaics, Forschungszentrum Jülich, Jülich, Germany

J. J. Jeronimo-Rendon, B. V. Lotsch

Nanochemistry Department, Max Planck Institute for Solid State Research, Stuttgart, Germany

R. Félix

Department Interface Design, Helmholtz-Zentrum Berlin für Materialien und Energie GmbH (HZB), Berlin, Germany

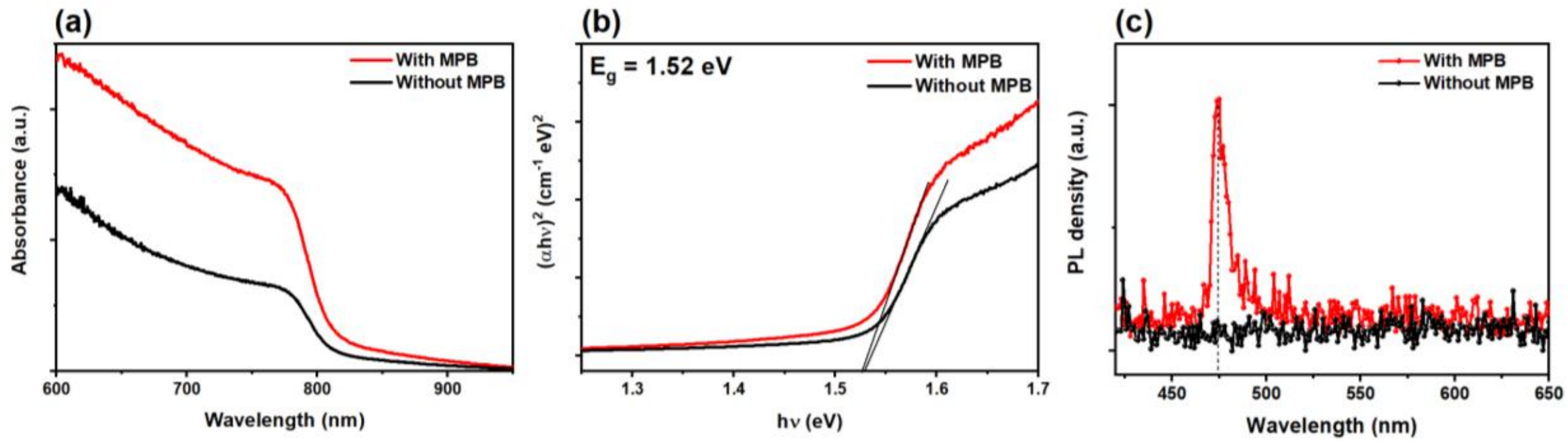

Figure S1. a) UV-vis absorbance, b) Tauc plot, and c) magnified PL spectra of the perovskite film, with and without MPB passivation.

Charge carrier lifetime calculation from TRPL spectra

The PL decay lifetime was obtained by fitting the TRPL spectra measured from the perovskite films with a biexponential decay function for the samples with and without MPB:

$$I(t) = A_1\, exp\left(-\frac{t}{\tau_1}\right) + A_2\, exp\left(-\frac{t}{\tau_2}\right) + K \qquad \text{(S1)}$$

where A_1 and A_2 are the decay amplitudes, τ_1 and τ_2 are the decay times, and K is a constant for the baseline offset. The average PL decay times (τ_{avg}) are further estimated using the following expression:

$$\tau_{avg} = \frac{A_1\tau_1^2 + A_2\tau_2^2}{A_1\tau_1 + A_2\tau_2} \qquad \text{(S2)}$$

Table S1. Amplitudes and lifetimes extracted from the bi-exponential fitting of the TRPL spectra.

**Sample**	$\boldsymbol{\tau_1}$ **(ns)**	$\boldsymbol{A_1}$ **(%)**	$\boldsymbol{\tau_2}$ **(ns)**	$\boldsymbol{A_2}$ **(%)**	$\boldsymbol{\tau_{avg}}$ **(ns)**
Without MPB	21	0.80	356	0.20	359
With MPB	52	0.43	708	0.57	1089

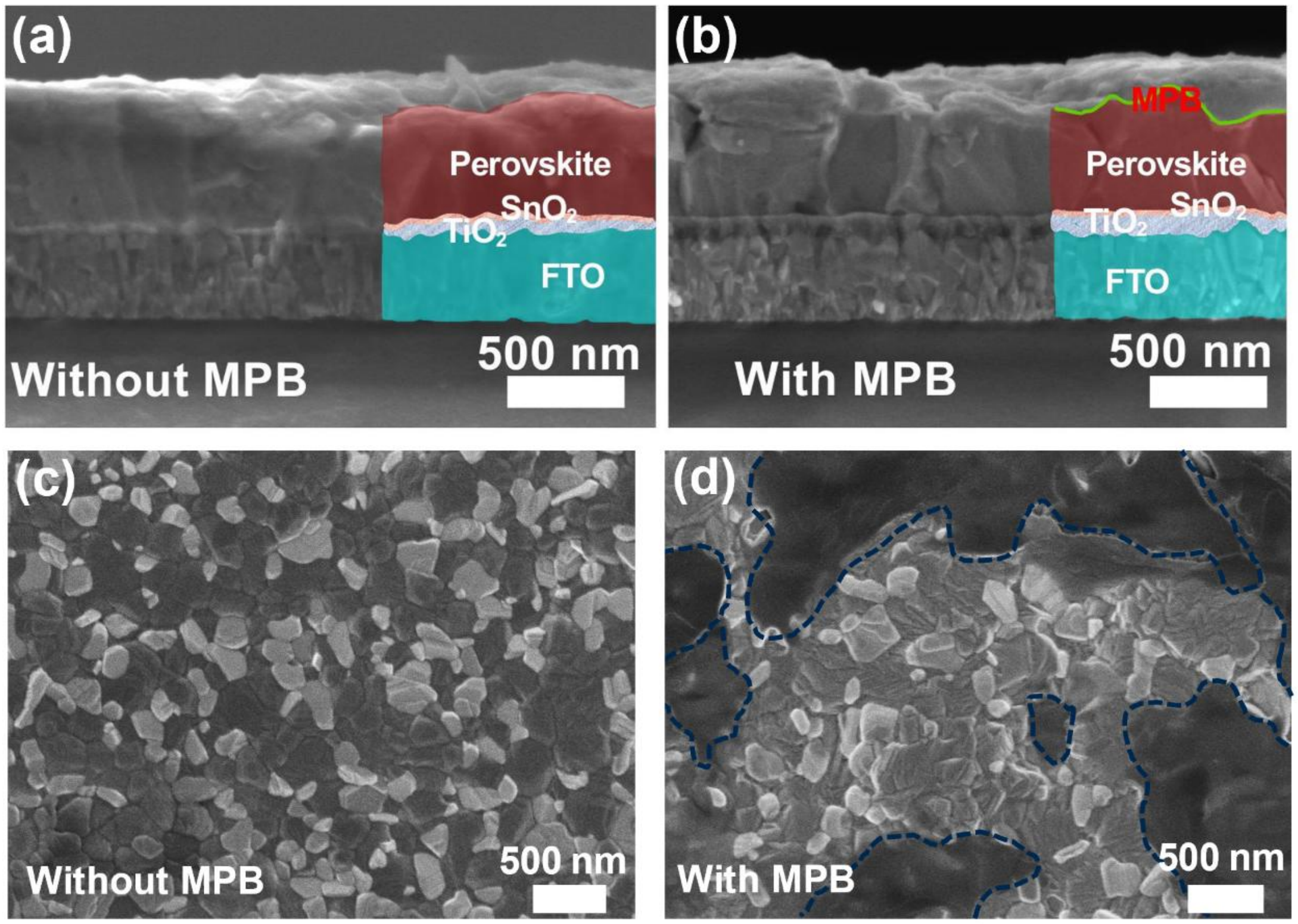

Figure S2. Cross-section and top-view SEM images of the FTO/ETL/perovskite films, a), c) without MPB passivation, b), d) with MPB passivation, respectively.

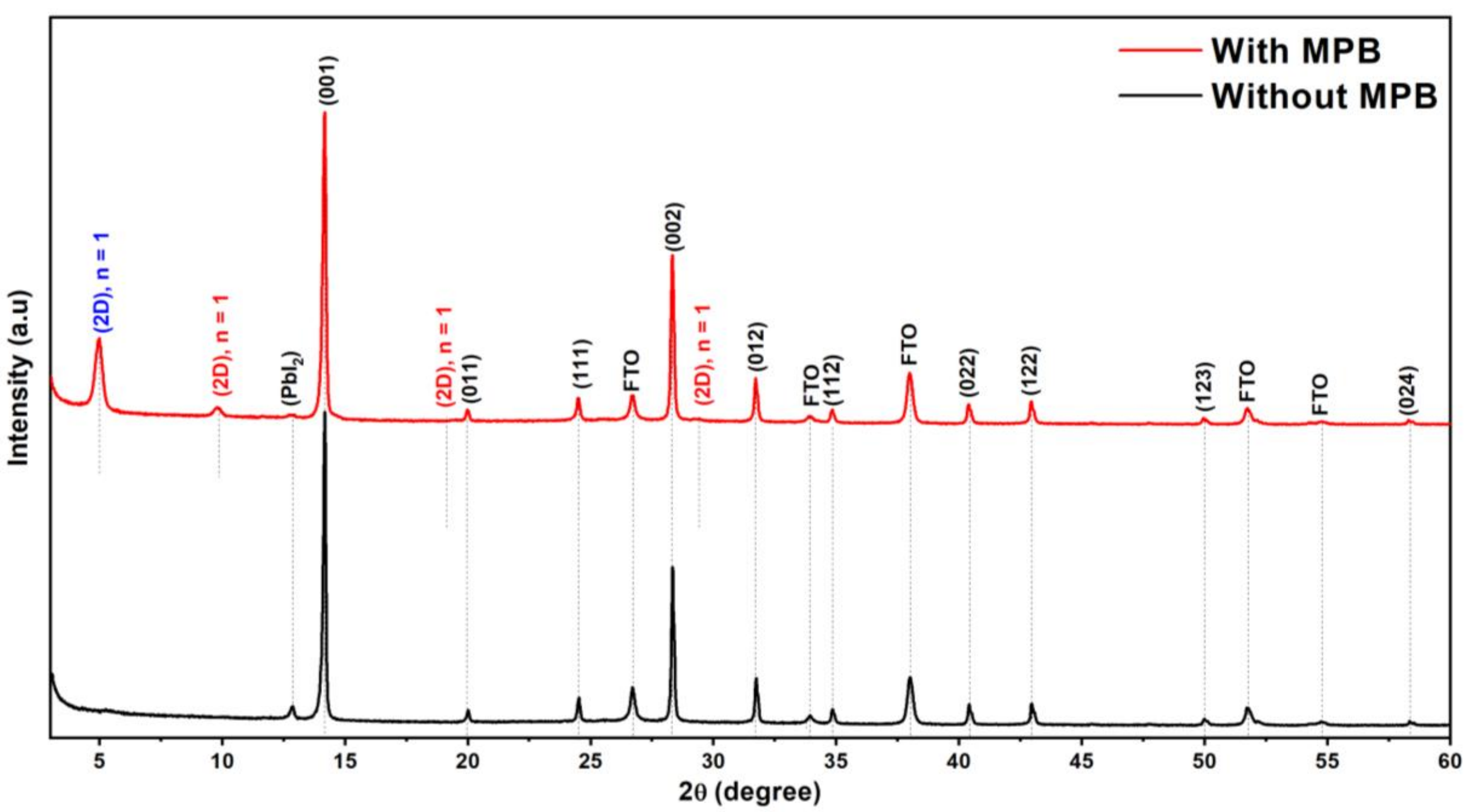

Figure S3. X-ray Diffraction (XRD) pattern for the perovskite film, without and with MPB passivation.

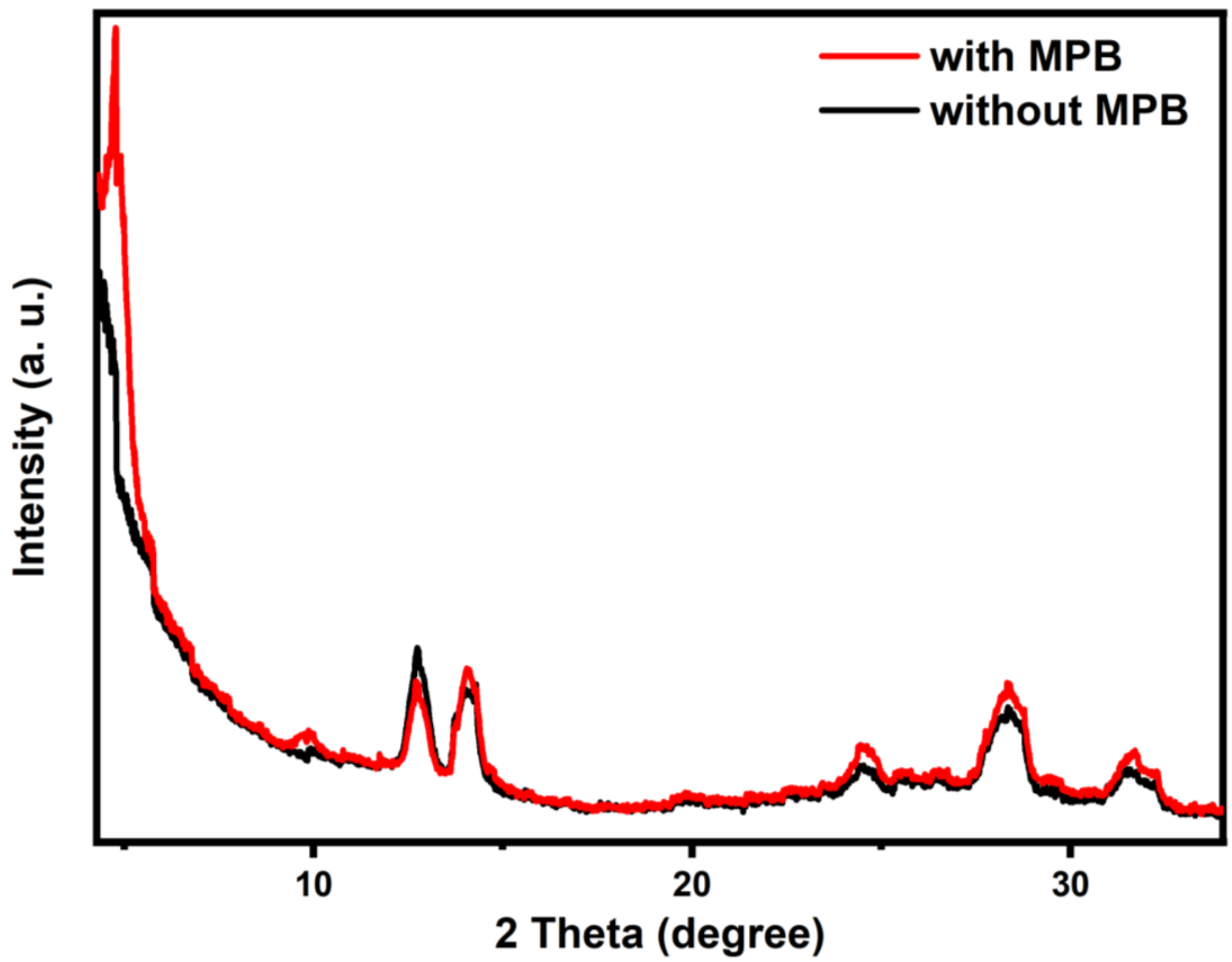

Figure S4. Integrated 1D GIWAXS profile of perovskite films without and with MPB treatment.

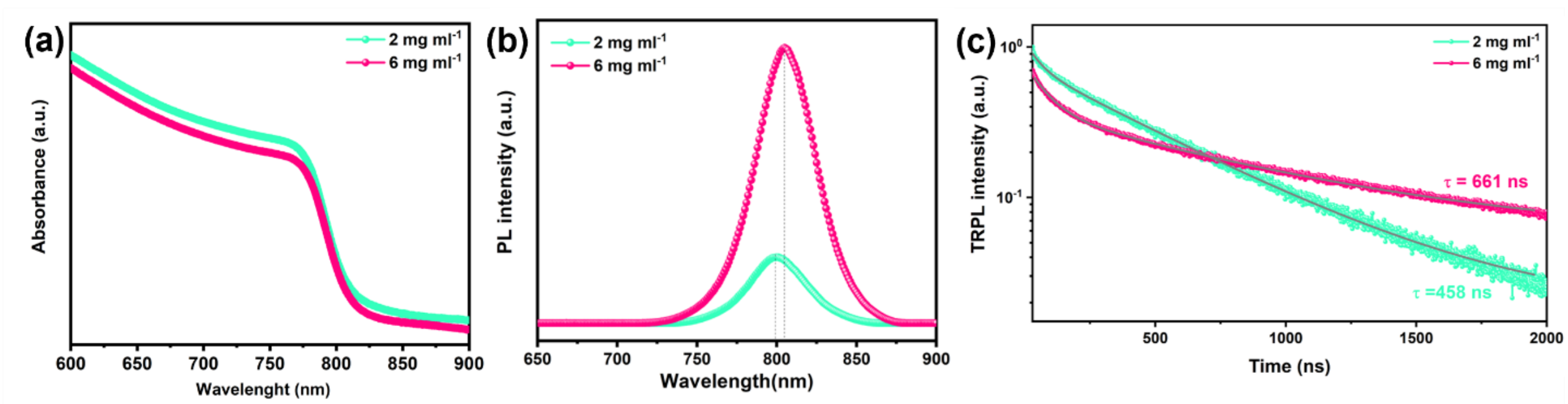

Figure S5. a) UV-Vis absorbance spectroscopy, b) steady-state PL, and c) TRPL for perovskite films with different concentrations of MPB passivation.

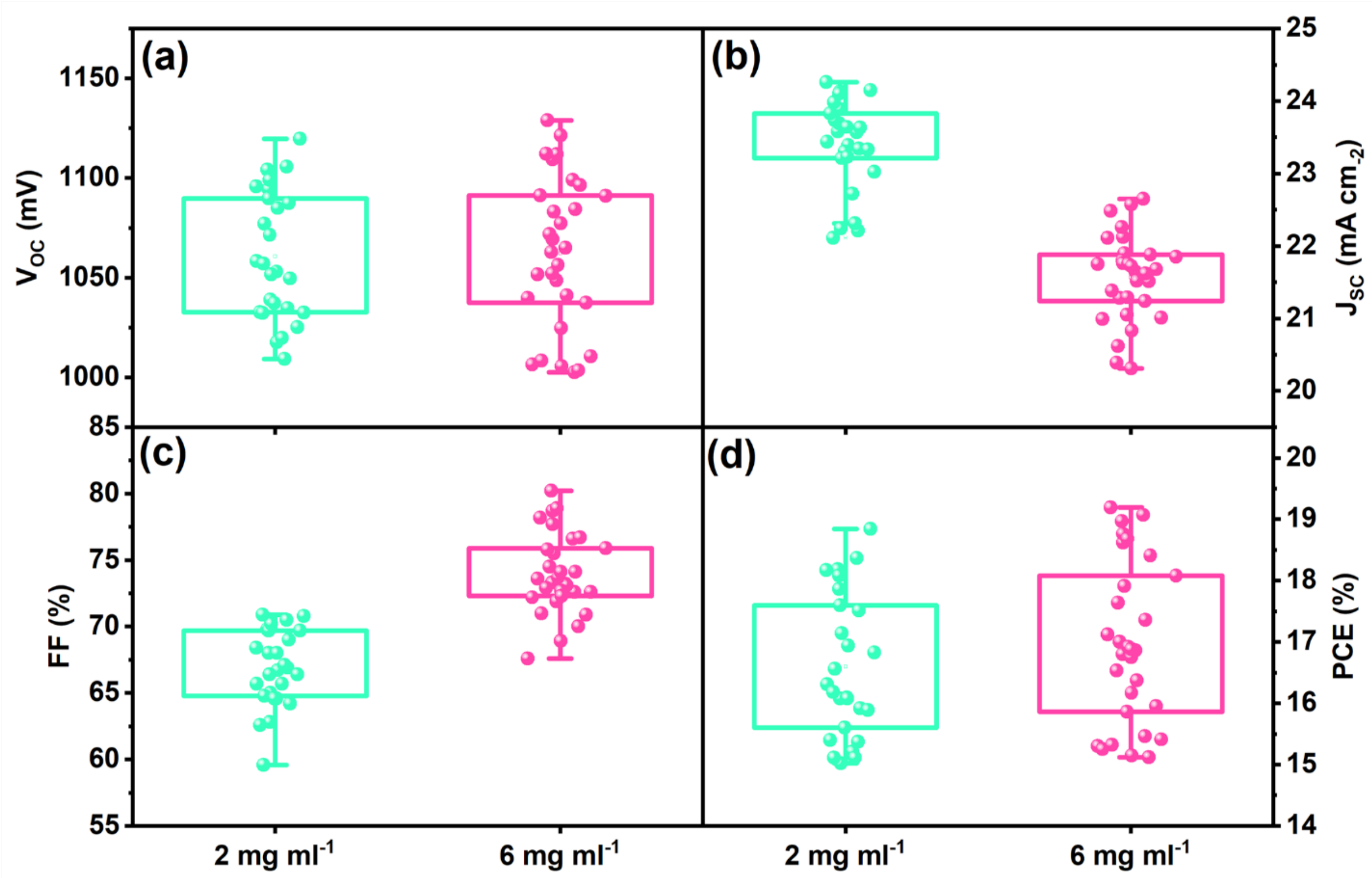

Figure S6. Statistical distribution of photovoltaic parameters, including a) V_{OC}, b) J_{SC}, c) FF, and d) PCE for PSCs with different concentrations of MPB passivation.

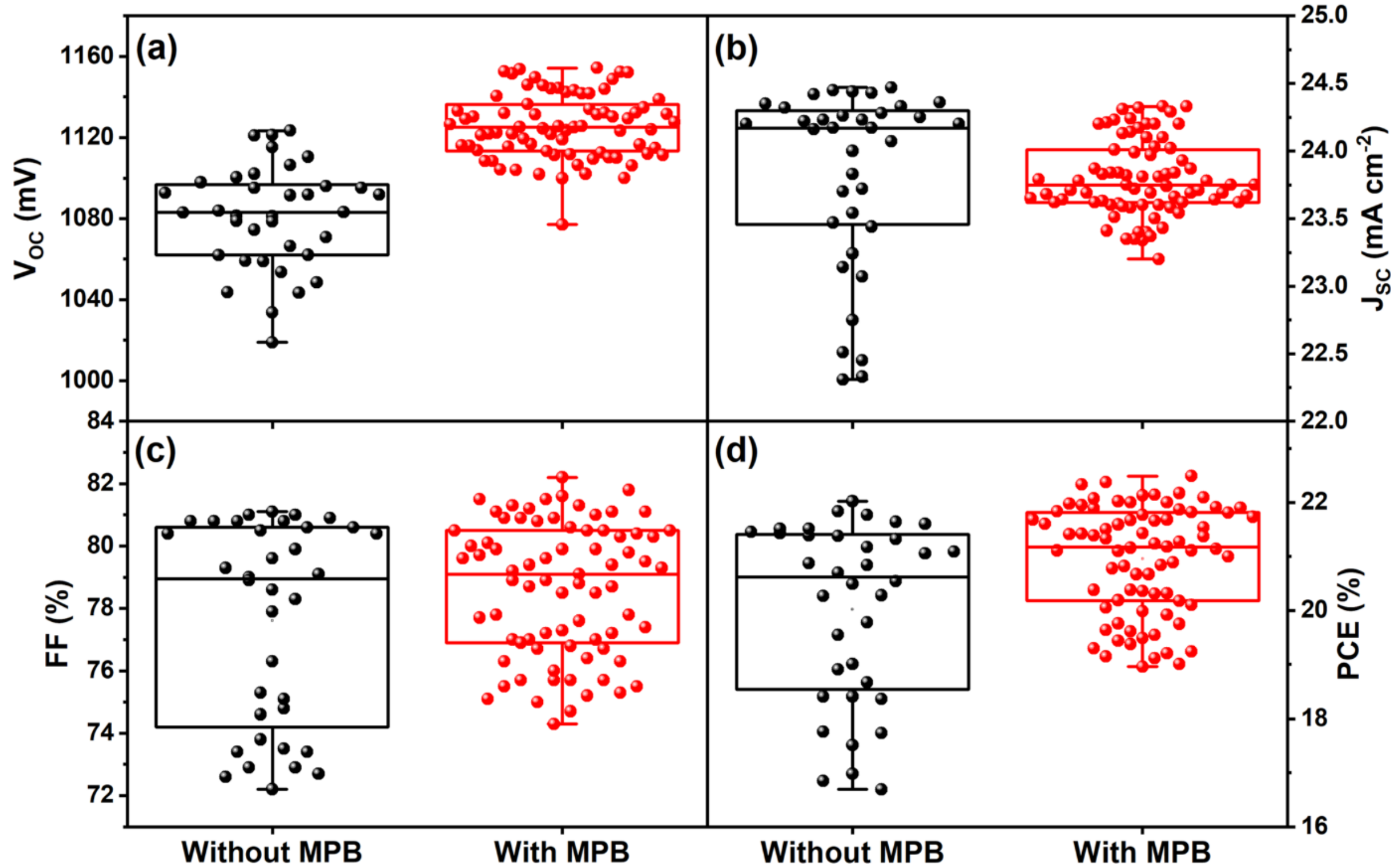

Figure S7. Statistical distribution of photovoltaic parameters, including a) V_{OC}, b) J_{SC}, c) FF, and d) PCE for 77 devices (per condition), fabricated from perovskite film, with and without MPB passivation.

Table S2. Photovoltaic parameters of champion devices passivated with MPB for forward and backward scan directions.

Passivation	PCE (%)	FF (%)	V_{OC} (mV)	J_{SC} (mA cm^{-2})
Forward	22.5	81.8	1144.0	24.02
Backward	22.7	82.3	1154.3	23.98

Table S3. Average photovoltaic parameters for 77 devices (per condition), fabricated without and with MPB, for the backward scan direction.

Passivation	V_{OC} (mV)	J_{SC} (mA cm^{-2})	FF (%)	PCE (%)
Without MPB	1081.0±25.6	23.60±0.74	77.6±3.2	20.0±1.6
With MPB	1125.8±16.3	23.80±0.29	78.6±2.1	20.9±0.9

FF loss analysis

Under the S-Q limit assumption, FF loss is mainly determined by non-radiative loss and charge-transfer loss. The higher the value of the maximum FF (FF_{max}), the lower the non-radiative recombination loss of the PSCs. FF_{max} can be calculated by the following equation:

$$FF_{max} = \frac{\frac{qV_{OC}}{K_BT} - \ln\left(\frac{qV_{OC}}{K_BT} + 0.72\right)}{\frac{qV_{OC}}{K_BT} + 1} \quad \text{(S3)}$$

Where K_B, T, and q denote the Boltzmann constant, the absolute temperature (300 K), and the elementary charge, respectively.

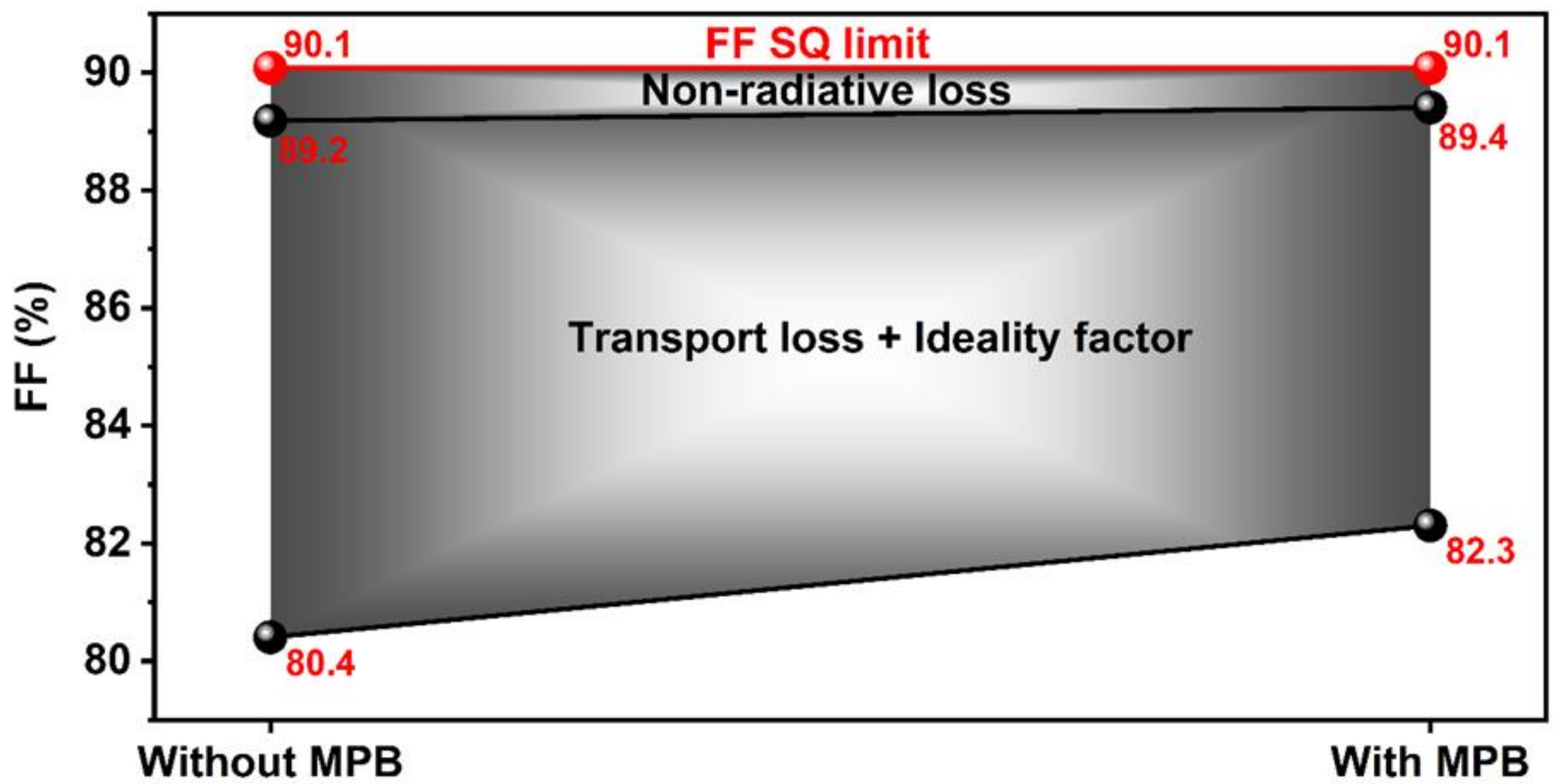

Figure S8. The FF loss analysis for PSCs without MPB and MPB treatment.

Methods for calculating carrier mobility, defect density, and diffusion length

The hole mobility and defect densities in the perovskite device were predicted by fabricating a hole-only device with a structure of FTO/2PACz/perovskite/spiro-OMeTAD/Au. The *J*-*V* response of the device was then recorded in the dark under an ambient atmosphere using a Keithley 2400. The double logarithmic plot of the result will contain a linear and a quadratic dependence of J on the applied bias V and a trap-filling transition (TFL). The linear one is the Ohmic response of the device. Trap density determines the voltage:

$$V_{TFL} = \frac{e n_{trap} L^2}{2\varepsilon_r \varepsilon_0} \tag{S4}$$

which at all traps are filled, $e = 1.602\times10^{-19}$ C is the elementary charge, n_{trap} is the trap density, L is the thickness of the film, $\varepsilon_0 = 8.854\times10^{-14}$ F cm^{-1} is the vacuum permittivity, ε_r is the relative dielectric constant.[1]

The quadratic part is the space-charge-limited current characteristic of the device, which is well described using a Mott-Gurney relation:

$$J = \frac{9\varepsilon_r\varepsilon_0\mu V^2}{8L^3} \qquad \text{(S5)}$$

where V, L, m, ε_0, and ε_r are the applied voltage, carrier mobility, vacuum permittivity, and the relative dielectric constant of perovskite, respectively.[2] The Mott-Gurney relation can be simplified as $J = bV^2$, with b = $\frac{9\varepsilon_r\varepsilon_0\mu}{8L^3}$, which is obtained from second-order polynomial fitting of the quadratic part of the curve. According to the literature, the dielectric constant ε_r of perovskite is determined to be 46.9. [3] The thickness of both perovskite films was 550 nm, which was verified by profilometry.

In addition, free carrier concentration can be calculated using the relation:

$$n_c = \frac{\sigma}{e\mu} \qquad \text{(S6)}$$

where σ is the conductivity extracted from the ohmic region of the *J-V* curve.

The carrier diffusion length was calculated from the relation:

$$L_D = \sqrt{\frac{\mu\tau k_B T}{e}} \qquad \text{(S7)}$$

where k_B is the Boltzmann constant, T is the absolute temperature, and τ is the hole carrier lifetime.

Table S4. The L_D, σ, n_c, V_{TFL}, n_{trap}, and μ_h values of space-charge-limited current (SCLC) measurements for hole-only devices, including without and with MPB passivation.

sample	L_D (μm)	Sigma (Ω^{-1} cm^{-1})	n_c (cm^{-3})	V_{TFL} (V)	n_{trap} (cm^{-3})	μ_h (cm^2 V^{-1} s^{-1})
Without MPB	0.54	4.24×10^{-8}	79.6×10^{10}	0.27	0.46×10^{16}	0.33
With MPB	0.77	7.14×10^{-8}	133.0×10^{10}	0.22	0.37×10^{16}	0.34

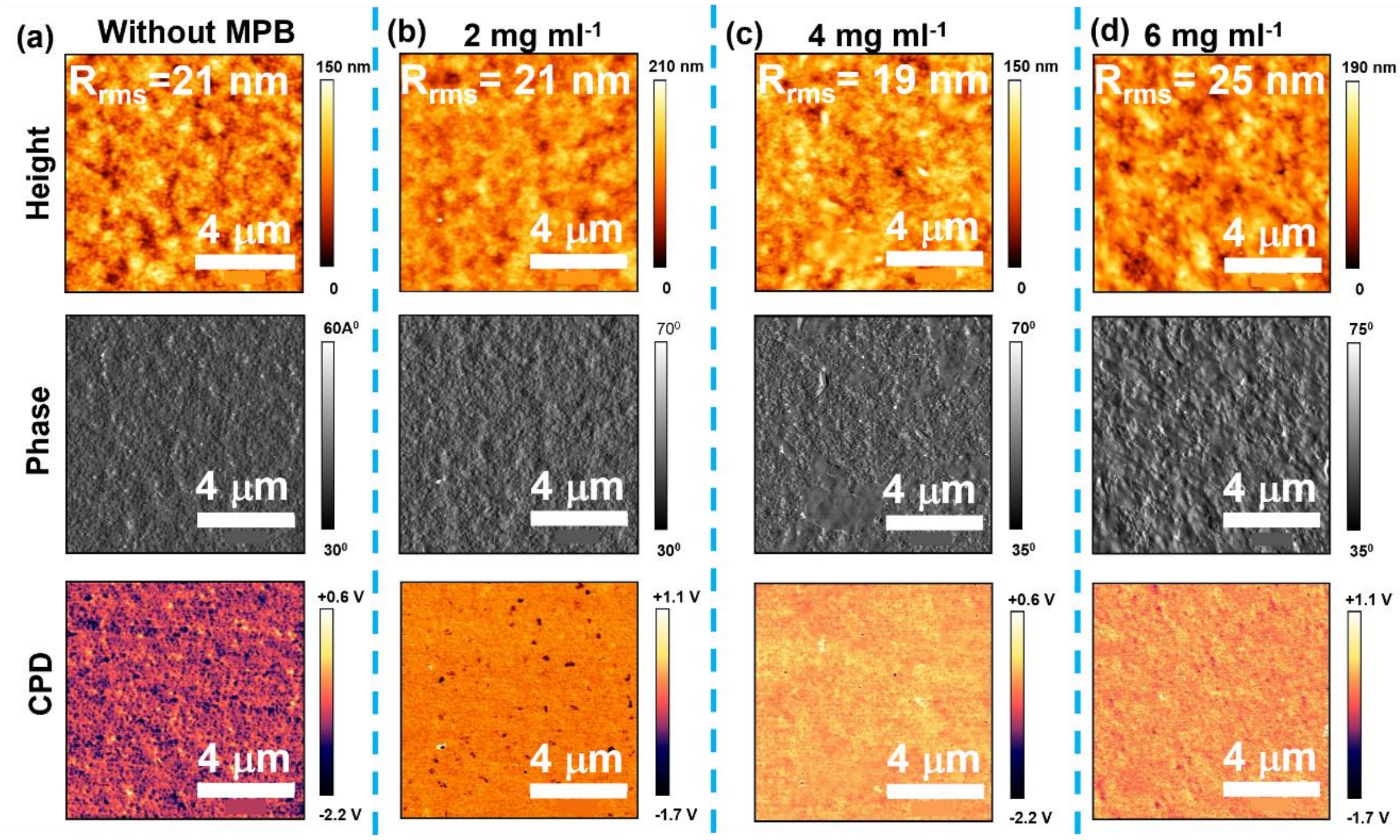

Figure S9. Kelvin Probe Force Microscopy (KPFM) analysis of surface potential for perovskite thin films: a) without MPB and with different concentrations of MPB passivation, including b) 2 mg ml^{-1}, c) 4 mg ml^{-1}, and d) 6 mg ml^{-1}.

Table S5. The roughness and CPD values of the KPFM measurement of perovskite films without MPB and with different concentrations of MPB passivation.

Sample	Height (nm)	CPD (mV)	
	RMS	Average	RMS
Without MPB	21	-1.02	0.25
With MPB (2 mg ml^{-1})	21	-0.36	0.19
With MPB (4 mg ml^{-1})	19	-0.35	0.13
With MPB (6 mg ml^{-1})	25	0.05	0.16

Table S6. Summary of reports on the application of dipole-moment passivation agents in PSCs.

Passivation agent	V_{OC} (V)	PCE (%)	Structure	Ref
4-MeO-PEAI	1.13	22.63	p-i-n	[4]
4-MeO-PEABr	1.17	20.31	n-i-p	[5]
4-MeO-PEA^+	1.10	19.64	p-i-n	[6]
PTFCN	1.05	21.50	n-i-p	[7]
O-Dipoles	1.14	22.18	n-i-p	[8]
4-FBAC	1.01	19.23	n-i-p	[9]
4-FAL	1.13	20.48	p-i-n	[10]
EAD4	1.14	20.00	p-i-n	[11]
PCBB-3N-3I	1.13	21.10	p-i-n	[12]
4-MeO-PEABr	1.15	22.7	n-i-p	Current work

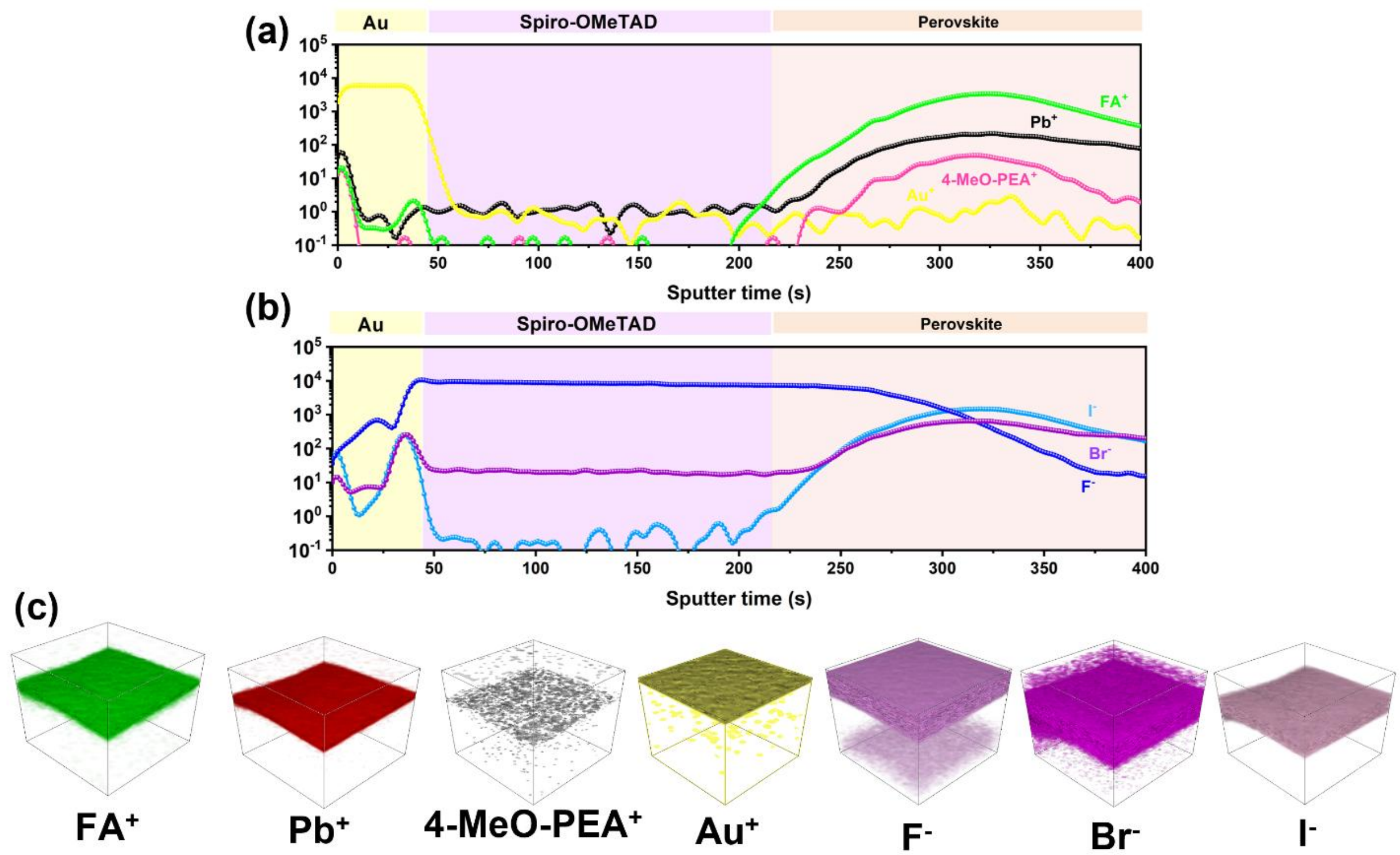

Figure S10. a), b) TOF-SIMS profile of the perovskite devices with MPB from the perovskite/4-MeO-PEABr/Spiro-OMeTAD/Gold, and c) TOF-SIMS 3D topography images of the different ions.

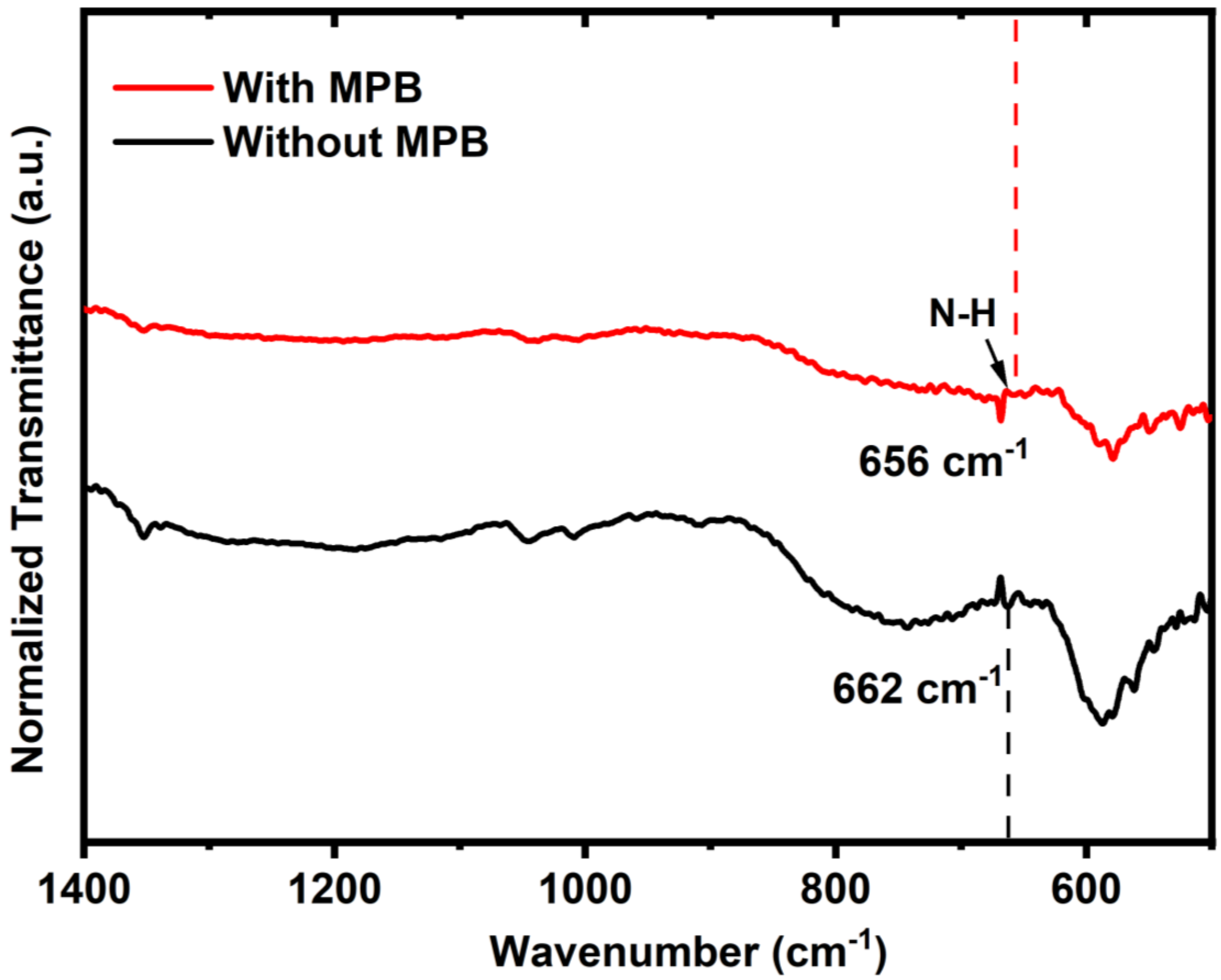

Figure S11. FTIR spectra (for low wavenumbers) of the perovskite films without and with MPB treatment.

References

[1] J. H. Heo, M. S. You, M. H. Chang, W. Yin, T. K. Ahn, S.-J. Lee, S.-J. Sung, D. H. Kim, S. H. Im, *Nano Energy* **2015**, *15*, 530.
[2] C.-H. Chiang, C.-G. Wu, *Nature Photon* **2016**, *10*, 196.
[3] Q. Yang, X. Liu, S. Yu, Z. Feng, L. Liang, W. Qin, Y. Wang, X. Hu, S. Chen, Z. Feng, G. Hou, K. Wu, X. Guo, C. Li, *Energy Environ. Sci.* **2021**, *14*, 6536.
[4] Z. Yang, J. Chen, M. Li, M. Qi, G. Zhang, R. Chen, J. Hu, X. Liu, C. Qin, L. Xiao, S. Jia, *Chemical Engineering Journal* **2024**, *494*, 152955.
[5] S. Zhang, L. Zhang, Q. Tian, X. Gu, Y. Du, K. Zhao, S. (Frank) Liu, *Advanced Energy Materials* **2022**, *12*, 2103007.
[6] F. Meng, X. Shang, D. Gao, W. Zhang, C. Chen, *Nanotechnology* **2022**, *33*, 065201.
[7] W. Wang, P. Chen, C. Chiang, T. Guo, C. Wu, S. Feng, *Adv Funct Materials* **2020**, *30*, 1909755.
[8] N. Guan, Z. Deng, K. Zou, Y. Liu, Y. Wang, X.-C. Yang, Z. Jiao, *ACS Appl. Mater. Interfaces* **2025**, *17*, 47649.
[9] W. Xu, Y. Zhang, Y. Huang, Y. Tao, J. Wang, W. Liu, D. Wang, Z. Zhang, J. Xiong, J. Zhang, *Small* **2025**, *21*, 2407063.
[10] S. Zhao, J. Xie, G. Cheng, Y. Xiang, H. Zhu, W. Guo, H. Wang, M. Qin, X. Lu, J. Qu, J. Wang, J. Xu, K. Yan, *Small* **2018**, *14*, 1803350.
[11] E. Aktas, N. Phung, H. Köbler, D. A. González, M. Méndez, I. Kafedjiska, S.-H. Turren-Cruz, R. Wenisch, I. Lauermann, A. Abate, E. Palomares, *Energy Environ. Sci.* **2021**, *14*, 3976.
[12] J. Huang, *Sci. China Chem.* **2020**, *63*, 145.
